# Long-Term Visual Quality after Microincision Cataract Surgery

**DOI:** 10.1155/2020/9318436

**Published:** 2020-09-15

**Authors:** Qing Huang, Ruili Li, Liwen Feng, Na Miao, Wei Fan

**Affiliations:** ^1^Department of Ophthalmology, West China Hospital of Sichuan University, Chengdu, Sichuan, China; ^2^Department of Medical Examination Center, The Second Affiliated Hospital of Chongqing Medical University, Chongqing, China

## Abstract

**Purpose:**

Few studies have focused on long-term postoperative visual quality. This study aimed to evaluate the long-term visual quality after microincision cataract surgery (MICS).

**Methods:**

96 patients (144 eyes) diagnosed with age-related cataracts were enrolled in this one-year study. The patients underwent MICS and received aspheric monofocal intraocular lens implants. Uncorrected distance visual acuity (UDVA) was evaluated together with best-corrected distance visual acuity (BCDVA), best-corrected near visual acuity (BCNVA), contrast sensitivity, and surgically induced astigmatism (SIA).

**Results:**

Compared to preoperative measurements, UDVA, BCDVA, and BCNVA were significantly better after surgery (*P* < 0.001), and they remained stable throughout follow-up. Contrast sensitivity was also significantly better after surgery (*P* < 0.001). Mean SIA during follow-up was 0.57 ± 0.33 D at 1 week, 0.36 ± 0.25 D at 3 months, and 0.18 ± 0.16 D at 1 year. SIA decreased significantly during the postoperative period (*P* < 0.001). The 1-year postoperative absolute residual diopter value was 0.32 ± 0.28 D.

**Conclusion:**

MICS can provide excellent visual quality as soon as on postoperative day 1, which persists during the follow-up period of 1 year. In contrast to previous studies, SIA decreases over time and may not completely stabilize for as long as 1 year postoperatively.

## 1. Introduction

Age-related cataract is the leading cause of visual impairment and blindness among elders [1]. Phacoemulsification has now become a mainstream treatment for cataracts [2]. Microincision cataract surgery (MICS), which is performed through an incision of less than 2 mm [3], has been developed as a method of minimizing corneal trauma and providing better postoperative outcomes than standard small incision phacoemulsification [4]. Significant improvements in phacoemulsification techniques and intraocular lens (IOL) technology have turned cataract surgery from an exceptional, sight-saving operation to a routine refractive procedure [5]. Optimally, vision-restoring surgery should increase both visual acuity and quality. Numerous clinical trials indicate that cataract surgery increases visual acuity, but it can also result in surgically induced astigmatism (SIA) [4, 6, 7], which limits improvements in visual quality. SIA is associated with many factors, including incision size [8], preoperative astigmatism, amount of manipulation during surgery [9], ultrasound technology, and instrumentation [3]. Numerous studies have reported lower SIA after MICS than after regular incision of 2.5–3.0 mm [2, 6, 10–12]. These studies suggest that MICS can reduce SIA, accelerate visual rehabilitation, and improve incision integrity. However, most previous studies on SIA involved follow-up of only 1 day to 6 months [2, 6, 10–13].

Few studies, particularly in the long term, have examined how MICS may affect SIA and visual quality like contrast sensitivity. Therefore, the aim of this study was to evaluate visual quality in terms of SIA up to 1 year after MICS.

## 2. Methods

### 2.1. Patients

This prospective observational clinical study enrolled a total of 96 patients (144 eyes) treated in the study between October 2015 and January 2016. The study protocol followed the guidelines of the Declaration of Helsinki and was approved by the Ethics Committee in our hospital. All patients were informed about the study and provided informed consent before participating.

### 2.2. Inclusion and Exclusion Criteria

Patients were enrolled consecutively if they were diagnosed with age-related cataracts of grades II-IV according to Emery-Little classification [14] using s slit-lamp microscope (Topcon SL-1E, Tokyo, Japan). Patients were excluded if (a) they had been diagnosed with ocular diseases, such as corneal disorders, glaucoma, active uveitis, and retinal or optical nerve diseases; (b) they had a corneal endothelial cell count of <1500 cells/mm [2]; (c) their axial length exceeded 26.5 mm; or (d) they had a history of ocular surgery or ocular trauma.

### 2.3. Preoperative Examinations

All patients underwent comprehensive systemic and ophthalmic examination including slit-lamp microscopy, ocular biometry (IOL Master 500; Carl Zeiss Meditec, Jena, Germany), ocular ultrasound, corneal topography, corneal specular microscopy, and optical coherence tomography (OCT). The following visual parameters were determined: uncorrected distance visual acuity (UDVA), best-corrected distance visual acuity (BCDVA), best-corrected near visual acuity (BCNVA), contrast sensitivity, and intraocular pressure (IOP).

### 2.4. Intraoperative Procedures

All eyes underwent the same MICS procedures using a Stellaris Phaco unit (Bausch & Lomb, USA). After topical anesthesia (Benoxil, 0.4% solution), a corneal incision of 2.0 mm (at around the 10 o'clock position) was made, followed by a second incision at around the 2 o'clock position. Continuous curvilinear capsulorhexis was made with a diameter of approximately 5.0–5.5 mm. After phacoemulsification of the nucleus and irrigation/aspiration of the remaining cortex, an Akreos MI60 IOL (Bausch & Lomb, USA) was inserted in the capsular bag using an injector system. All surgeries were performed by the same experienced surgeon (W. F.).

### 2.5. Follow-Up

Postoperative assessments were performed on the day after surgery as well as 1 week, 3 months, and 1 year afterwards. At each follow-up, detailed slit-lamp microscopy and corneal topography were performed, and the following parameters were determined: UDVA, BCDVA, BCNVA, contrast sensitivity, and IOP. SIA was determined using an SIA calculator (http://www.sia-calculator.com). Contrast sensitivity was measured using CSV-1000E (Vector Vision, USA). Anterior segment OCT (AS-OCT) (Carl Zeiss Meditec, Dublin, CA, USA) was performed at 1 week and 3 months postoperatively. Corneal specular microscope was measured preoperatively and at 3 months postoperatively.

### 2.6. Statistical Analysis

Statistical analysis was performed using SAS 9.2 software. For statistical analysis of visual acuity, logarithms of the minimum angle of resolution (logMAR) were used. Contrast sensitivity values were also log-transformed (Metrovision). Quantitative data were expressed as mean ± SD. Qualitative data were expressed as frequencies and percentiles. Preoperative and postoperative measurements were assessed for significance using the paired-samples *t*-test and the Wilcoxon signed rank test. Differences across time points were analyzed for significance using analysis of variance (ANOVA) followed by the Dunnet-*t*-test for differences between groups. Differences associated with *P* < 0.05 were considered statistically significant.

## 3. Results

Ninety-six patients (144 eyes) with age-related cataracts ranging in age from 60 to 85 years were enrolled in this study. Among them, 31 were men (47 eyes; mean age, 71.20 ± 10.08 years) and 65 were women (97 eyes; mean age, 68.71 ± 8.32 years). Mean axial length was 23.57 ± 1.00 mm (range: 21.57–26.17 mm). Patients with a cataract nucleus grade of II, III, or IV-V accounted for 21.57%, 66.67%, and 11.76%, respectively.

### 3.1. Visual Acuity and Quality Measurements

**Figure 1:**
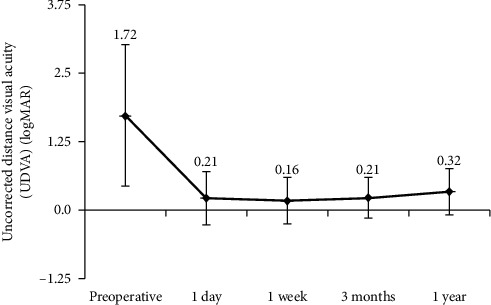
Preoperative and postoperative uncorrected distance visual acuity (UDVA) (*n* = 144 eyes).

Figure 1 shows preoperative and postoperative UDVA. Mean UDVA (in logMAR) was 1.72 ± 1.30 preoperatively, 0.21 ± 0.49 at postoperative day 1, 0.16 ± 0.42 at 1 week, 0.21 ± 0.37 at 3 months, and 0.32 ± 0.41 at 1 year. The preoperative value was significantly higher than all postoperative values (all *P* < 0.001). UDVA did not vary significantly during follow-up (*P* > 0.05).

**Figure 2:**
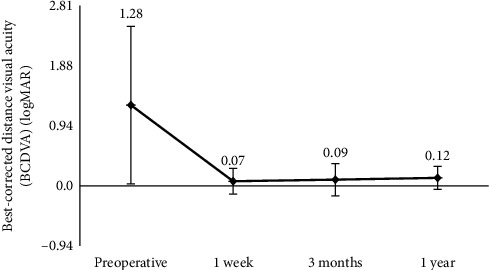
Preoperative and postoperative best-corrected distance visual acuity (BCDVA) (*n* = 144 eyes).

Figure 2 shows preoperative and postoperative BCDVA. Mean BCDVA (in logMAR) was 1.28 ± 1.24 preoperatively and then 0.07 ± 0.20 at 1 week, 0.09 ± 0.25 at 3 months, and 0.12 ± 0.18 at 1 year postoperatively. A mixed linear model showed significant differences between the preoperative value and every postoperative time point (*P* < 0.001). The value did not vary significantly during the postoperative period (*P* > 0.05).

The BCNVA was compared using the Wilcoxon signed rank test and found to be significantly better at every postoperative time point than preoperatively (*P* < 0.001; Table 1).

**Figure 3:**
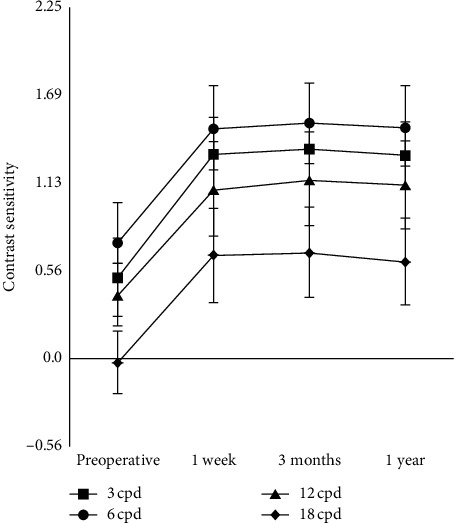
Preoperative and postoperative contrast sensitivity values (*n* = 144 eyes) (cpd, cycles per degree).

Figure 3 shows the preoperative and postoperative contrast sensitivity at spatial frequencies of 3, 6, 12, and 18 cycles per degree. For each spatial frequency, contrast sensitivity was significantly higher at every postoperative time point than preoperatively (*P* < 0.001), and it remained stable for up to 1 year postoperatively. Nevertheless, a decreasing trend was observed at 18 cycles per degree.

### 3.2. Surgically Induced Astigmatism, Corneal Thickness at the Incision, and Central Corneal Thickness

**Figure 4:**
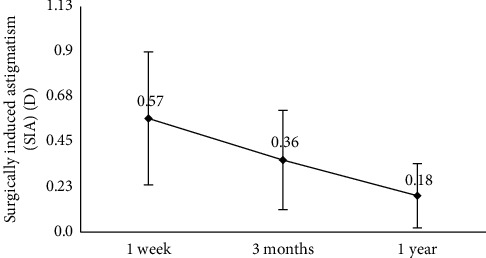
Variation in surgically induced astigmatism (SIA) during the follow-up period (*n* = 144 eyes).

**Figure 5:**
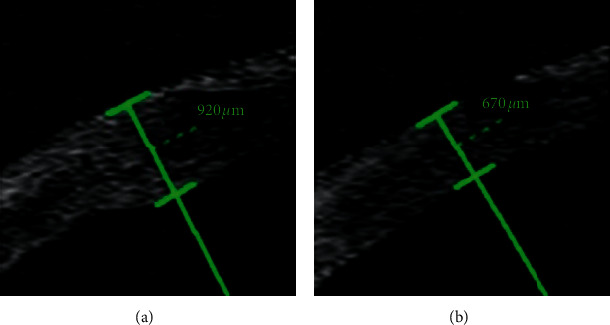
Representative anterior segment optical coherence tomography images showing corneal thickness measurements at the incision site at 1 week (a) and 3 months (b) postoperatively.

Patients in this study had a mean anterior corneal astigmatism of 0.80 ± 0.43 D (range, 0.29 to 1.87 D) preoperatively. After the surgery, the average SIA was 0.57 D (0.57 ± 0.33 D) at 1 week postoperatively and decreased significantly during the postoperative period (*P* < 0.001, Figure 4), with a mean value of 0.36 ± 0.25 D at 3 months. SIA was not stabilized and kept decreasing to 0.18 D (0.18 ± 0.16 D) by 1 year postoperatively. A significant corneal edema at the incision (mean corneal thickness 861.71 ± 125.71 *μ*m was seen at 1 week (Figure 5(a)) and a near normal thickness of cornea at the site of incision (mean 686.83 ± 53.17 *μ*m, *P* < 0.001) at 3 months (Figure 5(b)). The mean central corneal thickness was 546.79 ± 40.81 *μ*m at 1 week and 538.61 ± 39.57 *μ*m at 3 months (*P*=0.042).

### 3.3. Residual Refractive Power

The 1-year mean value of absolute postoperative residual diopter value was 0.32 ± 0.28 D, compared to preoperative target diopter value of −0.08 ± 0.12 D (absolute preoperative target diopter value of 0.12 ± 0.09 D).

### 3.4. Intraoperative and Postoperative Complications

No major intraoperative complications such as posterior capsule rupture happened in any of the cases. Mean corneal endothelial cell density was 2475.77 ± 315.17 cells/mm^2^ preoperatively and 2278.83 ± 445.15 cells/mm^2^ at 3 months postoperatively (*P* < 0.001). Mean endothelial cell count loss was 7.76 ± 13.22% at 3 months postoperatively. Three of the patients showed Descemet's membrane detachment by anterior segment OCT at postoperative day 1, which resolved spontaneously on subsequent examination. All incisions were self-limited by 3 months postoperatively. At 3-month follow-up, 3 eyes (2.08%) required neodymium : YAG laser capsulotomy due to anterior capsule contraction. At 12-month follow-up, posterior capsular opacification was seen in 7 eyes (4.86%), for which mean BCDVA was 0.047 ± 0.03 logMAR. None of these eyes required neodymium : YAG laser capsulotomy.

## 4. Discussion

This study evaluated visual quality over a period of 1 year after MICS and Akreos MI60 IOL implantation. The data showed significant improvement in UDVA, BCDVA, and BCNVA (Figures 1 and 2 and Table 1). Visual acuity increased significantly within 1 week and remained stable for 1 year postoperatively. A study found that the increase in epithelial thickness and CCT was correlated with CDVA at a near term after cataract surgery [15]. The statistically significant difference of CCT between 1 week and 3 months (*P*=0.042) did not seem to affect UDVA and BCDVA at an early time postoperatively. CCT postoperatively was influenced by many factors including phaco time, phaco power, and age [15, 16]. Further studies are needed to explore the relationship between CCT and visual acuity. Moreover, the SIA was small (0.36 ± 0.25 D) at 3 months postoperatively. These results were in line with expected visual outcomes after MICS.

Contrast sensitivity after phacoemulsification has been studied for follow-up periods shorter than one year [6, 17, 18]. Espíndola et al. [19] reported improved contrast sensitivity after phacoemulsification implantation of an aspherical monofocal IOL with a spherical surface lens, especially at low and intermediate spatial frequencies. Similarly, we found that 2.0 mm microincision phacoemulsification with aspheric monofocal IOL implantation significantly improved contrast sensitivity at low and intermediate spatial frequencies of 3, 6, and 12 cycles per degree between 1 week and 1 year postoperatively. Contrast sensitivity was also significantly increased at 18 cycles per degree, although there was a decreasing trend by 1 year postoperatively (Figure 3). This may have been caused by posterior capsule opacity, which nevertheless did not cause significant hazy eyesight.

SIA occurs when cataract surgery destroys corneal integrity and is determined by the biomechanical properties of the cornea as well as the incision location, shape, length, type, and suture [20–23]. Small incisions cause significantly greater SIA than microincisions [10, 21]. In our study, mean SIA was 0.18 ± 0.16 D at 1-year follow-up, which is well below the value of 0.5 D, which is considered the upper limit of corneal astigmatism after successful refractive cataract surgery [24]. This may reflect the advantages of MICS and the fact that IOLs in our study were implanted through incisions of only 2.0 mm long.

One of the most interesting findings from our study was the timing of stabilization for SIA. Previous studies of SIA after coaxial MICS was followed up for fewer than 3 months postoperatively [6, 25], but SIA is not static: it has been shown to peak soon after surgery and subsequently decrease over time [13]. Indeed, we found that SIA fell significantly between one week and 3 months (Figure 4), corresponding to the significant remission of corneal edema (Figure 5), and it continued to decrease to as low as 0.18 ± 0.16 D up to 1 year postoperatively. This is in contrast with studies reporting that SIA remained constant between 1 month [13] and 3 months postoperatively [26]. SIA may decrease as a result of cell migration, restoration of the endothelial barrier, remission of edema that restores corneal curvature [23], and other factors such as changes in corneal biomechanic properties [27, 28]. It may take longer for SIA to stabilize than previously reported. Although further study should examine the timing of SIA stabilization, one thing is clear that the SIA from MICS is low enough for refractive cataract surgery and thus makes MICS advantageous to other procedures for patients with premium IOL implantation. The limitation in our study was the lack of anterior segment OCT at 1 year, although the thickness of the cornea at the site of incision almost returned to normal at 3 months.

## 5. Conclusion

Our study of MICS suggests that the procedure is safe and effective and can lead to stable outcomes for at least 12 months postoperatively. Improvement in visual acuity and contrast sensitivity occurred soon after surgery and persisted throughout follow-up. On contrary to previous studies, SIA decreased over time and had not stabilized even by 1 year postoperatively.

## Figures and Tables

**Table 1:** Best-corrected near visual acuity (BCNVA) before and after surgery.

Jaeger	Preoperative (*N*)	Postoperative (*N*)		
		1 week	3 months	1 year
**J** ^*∗*^	13	0	0	0
**J1**	2	27	42	24
**J2**	6	78	61	76
**J3**	13	27	28	31
**J4**	26	8	10	13
**J5**	44	4	3	0
**J6**	18	0	0	0
**J7**	22	0	0	0
**Total**	144	144	144	144
**P**	—	<0.001	<0.001	<0.001

^*∗*^Helplessness of vision correction.

## Data Availability

The data used to support the findings of this study are available from the corresponding author upon request.
